# An Ethereum-Based Distributed Application for Enhancing Food Supply Chain Traceability

**DOI:** 10.3390/foods12061220

**Published:** 2023-03-13

**Authors:** Evripidis P. Kechagias, Sotiris P. Gayialis, Georgios A. Papadopoulos, Georgios Papoutsis

**Affiliations:** 1 Sector of Industrial Management and Operational Research, School of Mechanical Engineering, National Technical University of Athens, 15780 Athens, Greece; 2 Athens MBA Postgraduate Program, Athens University of Economics & Business and National Technical University of Athens, 11362 Athens, Greece

**Keywords:** blockchain, Ethereum, application, case study, food, supply chain, traceability, olive

## Abstract

In today’s era, humanity has been overwhelmed by technological revolutions that have changed and will continue to change how business operations are performed, directly or indirectly. At the same time, the processes within the supply chain are quite complex, and as technology and processes evolve, they become more and more challenging. Traceability has become a critical issue in the food industry to ensure safety, quality, and compliance with regulations. The adoption of blockchain technology in the food supply chain has gained significant attention as a potential solution to improve traceability. This paper presents the development of a distributed application for table olives’ traceability on the Ethereum network. The paper also presents a methodological framework, which can help anyone aiming to implement an Ethereum decentralized application and demonstrates the practical use of the developed application by a Greek table olives producer. The application significantly improved the producer’s product traceability by providing a secure, transparent, and efficient solution for tracking and tracing the products in the supply chain. The app reduced the time, increased the accuracy and reliability of data, improved supply chain efficiency, and helped the producer comply with international regulations and standards.

## 1. Introduction

Food traceability is the ability to track the movement of foods through the supply chain, from their origin, the stage of primary production, to their destination, the stage of consumption. Traceability is vital in the food supply chain, as it helps ensure food safety, quality, and compliance with regulations. Traceability is crucial in ensuring the food supply chain’s security [1]. If a food product is contaminated, traceability can help identify the source of the contamination and allow for targeted recall or removal of the contaminated products from the supply chain. This can help prevent the spread of foodborne illnesses and reduce the risk of public health crises. Traceability can also help ensure the quality and authenticity of food products. By tracking the movement of a product through the supply chain, it is possible to identify any potential issues that may affect the quality of the product, such as mishandling, storage, or transportation. This can help ensure that food products are of the highest quality when they reach the consumer [2].

Additionally, traceability is often a legal requirement for food products, with many countries having regulations in place that require food companies to be able to trace their products through the supply chain. Compliance with these regulations is essential for businesses to operate legally and avoid fines or legal issues. It can also help build trust between consumers and food companies. Consumers are increasingly concerned about the safety and quality of the food they consume, and being transparent about the supply chain can help build consumer confidence in a brand. Finally, traceability can help improve the efficiency of the supply chain [3]. By identifying potential issues in the supply chain, such as delays or bottlenecks, companies can make changes to improve the flow of products through the supply chain, reducing waste and optimizing delivery times. Overall, traceability is crucial for the food supply chain and can help ensure food safety, quality control, compliance, consumer trust, and supply chain efficiency. Without traceability, the food supply chain would be vulnerable to contamination, fraud, and inefficiency, which could have significant public health and economic consequences [4].

However, the current system of tracing food products is often inefficient, fragmented, and prone to errors. There is no universally accepted system for food traceability, which can make it difficult for companies to implement traceability across their supply chains. Companies may use different technologies and methods for tracking their products, making it challenging to share data and collaborate effectively. The food supply chain is often complex and involves multiple stakeholders, from farmers and producers to distributors and retailers. This complexity can make it challenging to trace a product’s origin and track its movement through the supply chain. Traceability requires collecting, managing, and sharing large amounts of data. Ensuring these data are accurate, complete, and up-to-date can be challenging, particularly when working with multiple stakeholders [5].

Blockchain technology, one of the most recent technological innovations for business processes, has the potential to offer a promising solution for improving the transparency and traceability of the food supply chain [6]. Blockchain is a distributed ledger that records transactions securely, imminently, and transparently, providing a tamper-proof and auditable record of events. Blockchain technology can provide a transparent view of the entire food supply chain, enabling all stakeholders to track the movement of food products from the farm to the consumer. This increased transparency can help build consumer trust and confidence in the food supply chain. Blockchain technology can help streamline the food supply chain by eliminating the need for intermediaries and reducing paperwork and bureaucracy. This can help reduce costs and improve the efficiency of the supply chain [7,8]. Blockchain technology can also provide a secure and tamper-proof record of all transactions, making it easier to trace the origin of food products and track their movement through the supply chain. This can help reduce the risk of food fraud, contamination, and counterfeiting. Moreover, by enabling faster and more accurate tracing of food products, blockchain technology can help improve food safety [9,10]. In the event of a food safety issue, blockchain can help identify the source of the problem quickly, enabling prompt action to be taken to minimize the impact on public health. Finally, blockchain technology can enable greater accountability in the food supply chain by providing a transparent and immutable record of all transactions. This can help ensure that all stakeholders in the supply chain are held accountable for their actions [11,12].

There is a growing interest in the potential of blockchain technology for improving traceability in the food supply chain. However, despite this interest, there is still a gap in research on the specific applications of blockchain for the traceability of specific commodities such as table olives [13]. While some pilot projects and initiatives are exploring the use of blockchain in the food industry, there is a lack of systematic research on the challenges, opportunities, and implications of implementing blockchain-based solutions for table olive traceability. This research gap presents an opportunity for further investigation and exploration of blockchain technology for the table olive industry. Several factors may contribute to this research gap. One factor is the relative novelty of blockchain technology, which is still a developing area with many unknowns and uncertainties [14]. Additionally, there may be limited resources and funding available for research on blockchain applications for table olive traceability, particularly in comparison to other research areas. Despite these challenges, the potential benefits of blockchain for the table olive industry are significant, and further research is needed to explore the feasibility, scalability, and impact of blockchain-based solutions for table olive traceability [15].

In this context, this paper presents an application of blockchain technology and, more specifically, the development of an Ethereum-based distributed application (dApp) that can help streamline and automate the traceability of the table olives supply chain, providing a secure and transparent way of recording transactions and ensuring that all stakeholders are held accountable for their actions. This research is the first of its kind for the table olives supply chain and manages to connect research with practice. The developed application is easy to use and offers a cost-effective traceability solution that is tested in real life at a Greek table olives producer. More specifically, the second section presents current research on blockchain applications for the food industry. The third section analyzes the table olives supply chain by also exploring the challenges and the potential benefits of blockchain traceability applications. The fourth section presents the materials and methods used for creating the Ethereum-based traceability distributed application. The fifth section demonstrates the case study of the research, i.e., the practical use of the table olives traceability application at a Greek table olives producer. Finally, the sixth section elaborates on the results of the practical application and explores the benefits gained.

## 2. Blockchain Applications in the Food Industry

Applications of blockchain technology for traceability in the food industry have significantly expanded during the last five years and reflect blockchain’s potential to improve transparency and trust in the food supply chain. Numerous research papers have been published in recent years exploring the use of blockchain technology for food traceability [16]. These papers generally recognize the potential of blockchain to enhance food safety and supply chain management, promote transparency and trust, and reduce the risk of fraud and foodborne illness. However, the research on blockchain applications in the food industry is still in its early stages, and several challenges and limitations need to be addressed. Some of the key challenges include the need for collaboration and standardization, the complexity of the technology, and the regulatory and legal barriers to implementation [8,12].

Despite these challenges, several ongoing initiatives and projects are exploring the use of blockchain in the food industry. These projects include pilot programs that use blockchain to track food products from farm to table, as well as research initiatives that aim to address the technical, economic, and regulatory issues associated with blockchain adoption. In their systematic review, Feng et al. (2020) explored the current state of research on blockchain technology in the agri-food sector. They identified the potential benefits of blockchain, such as enhancing food traceability, improving supply chain management, and promoting transparency and trust. However, they also highlighted the challenges associated with blockchain adoption, including technical issues, regulatory barriers, and the need for collaboration and standardization. The study concluded by identifying research gaps and proposing areas for future research, such as exploring the impact of blockchain on food safety and sustainability [12]. Vu et al. (2021) conducted a systematic literature review for blockchain adoption in food supply chains to investigate the perceptions, attitudes, barriers, and applications of blockchain technology. The study found that there is a high level of interest in blockchain but also a need for more outstanding education and awareness about its potential applications. The authors also identified the need for collaboration and standardization in order to achieve widespread adoption of blockchain in the food industry [17].

In a similar vein, Krithika (2022) conducted a survey on the use of blockchain in agriculture, including case studies from various countries and crops. The study found that there is a high level of interest in blockchain, particularly in relation to food traceability and supply chain management. However, the authors also identified several barriers to adoption, including the lack of technical expertise and the high costs associated with blockchain implementation [18]. Lin et al. (2019) proposed a blockchain-based traceability system for food safety that utilizes smart contracts and a decentralized network to record and track the movement of food products. The authors described the architecture of the system and the process of creating and verifying transactions. They also discussed the system’s benefits, such as improving transparency and trust in the food supply chain, as well as the implementation challenges, such as the need for standardization and interoperability [19]. Jaya et al. (2021) proposed a blockchain-based system for fishery products’ traceability that utilizes the Hyperledger Fabric blockchain to track the movement of seafood products from ocean to table. The authors performed several tests and achieved very efficient results in terms of performance and effectiveness. The authors also described the system’s benefits, such as improving food safety and reducing fraud [20].

Moreover, Naveen et al. (2022) proposed a blockchain-based traceability system for tea powder based on Hyperledger Fabric. The authors described the architecture of the system and the process of creating and verifying transactions. They also discussed the system’s benefits, such as improving transparency and trust in the tea supply chain. Duan et al. (2020) conducted a content-analysis-based review of the literature on the application of blockchain in the food supply chain. The authors identified the potential benefits of blockchain, such as enhancing traceability, improving supply chain management, and reducing food fraud. They also highlighted five possible obstacles, including a limited comprehension of blockchain, technological complexities, the manipulation of raw data, challenges in engaging all stakeholders, and inadequate regulatory frameworks [21]. Niya et al. (2020) proposed a blockchain-based system for Swiss dairy traceability through a decentralized dairy product Supply Chain Tracing (SCT) system. This system was created in collaboration with dairy producers and is designed to overcome the limitations of traditional and centralized SCT approaches. They also discussed the system’s benefits, including social aspects and risks [22]. Rünzel et al. (2021) designed a sustainable blockchain-based honey supply chain. The authors presented the development and implementation of an open and image-based honey traceability system on a global scale, including various technologies such as data-driven honey yield prediction and verification, a blockchain-based smart distribution system, and pollen signature verification through machine learning algorithms, along with a final customer information portal. When integrated, these technologies can address several United Nations’ Sustainable Development Goals [23].

Similarly, Yang et al. (2021) proposed a blockchain-based traceability system for vegetables and fruits that utilizes blockchain technology to distribute all required traceability information throughout the supply chain safely. Additionally, a smart contract based on reputation was developed to motivate network nodes to upload traceability data. Moreover, the authors conducted a performance analysis and practical application, with results indicating that their system enhances query efficiency, secures private information, ensures the authenticity and reliability of data in the supply chain management, and meets the necessary application requirements [24]. Rejeb (2018) proposed a traceability system for halal meat supply chains based on the integration of the Hazard Analysis and Critical Control Points (HACCP) method, blockchain technology, and Internet of Things (IoT) devices. The system involves collecting data from IoT devices at different stages of the supply chain, which are then stored on a blockchain-based platform for secure and immutable record keeping. The paper also discusses the implementation of the system and evaluates its effectiveness in improving supply chain traceability and quality control [25]. Adamashvili et al. (2021) proposed a wine supply chain based on blockchain technology that leverages the benefits of blockchain technology to address issues such as product provenance, anticounterfeiting, and traceability in the wine industry. The authors presented a model that integrates blockchain technology to simulate the operations of the supply chain. The results showed that blockchain technology enhances transparency, improves the efficiency of the wine supply chain, and creates a better wine consumption experience for customers. The authors argued that the proposed model can potentially benefit stakeholders across the wine supply chain, including wine producers, distributors, and consumers, by improving supply chain management, reducing fraud and counterfeit products, and providing a reliable and trustworthy method for wine traceability [26].

Several pilot programs have also been initiated to explore the use of blockchain in the food industry. Walmart, JD.com, IBM, and Tsinghua University National Engineering Laboratory for E-Commerce Technologies formed in 2017 a Blockchain Food Safety Alliance aimed at improving pork meat tracking, traceability, and safety in China. The companies will work together to establish a standards-based method of collecting data on pork origin, safety, and authenticity using blockchain technology to provide real-time traceability throughout the supply chain. The collaboration will provide accountability and greater transparency into how food is handled, from the farm to the consumer, and will work with food supply chain providers and regulators to develop the necessary standards, solutions, and partnerships to enable a broad-based pork safety ecosystem in China. The insights gained from this initiative will help improve processes such as recalls and verifications and enhance consumer confidence worldwide [27]. Nestlé partnered with OpenSC, a blockchain platform that enables consumers to track their food to its origin, in 2019, aiming to achieve new levels of supply chain transparency. In its quest for total transparency, Nestlé is the first major food and beverage company to pilot open blockchain technology. The pilot program will initially trace milk from farms in New Zealand to Nestlé factories and warehouses in the Middle East, followed by testing the technology using palm oil sourced from the Americas. These trials will help Nestlé determine the system’s scalability [28]. In collaboration with software provider SAP, Coke One North America (CONA), the Information Technology (IT) company in charge of Coca-Cola’s North American manufacturing supply chain processes, launched a pilot project in 2019 based on blockchain technology to enhance transparency and efficiency. The project was tested with two bottlers, Coca-Cola United and C.C. Clark, and is intended to be implemented across 70 manufacturers who collectively transport more than 160,000 bottles of Coke each day [29].

More recently, in 2022, Unilever and SAP collaborated on a pilot project that uses blockchain technology to increase transparency and accountability in the palm oil supply chain. The project aims to support sustainable and deforestation-free palm oil production by providing real-time visibility of the supply chain and tracing the origin of palm oil from the plantation to the refinery. The project involves tracking and verifying the sustainability practices of palm oil smallholders and suppliers, allowing Unilever to make more informed sourcing decisions and reduce the risk of purchasing palm oil linked to deforestation [30]. Carrefour started using blockchain with its own branded “Carrefour Bio” organic products in 2022 to satisfy consumers’ increasing demand for transparency in producing and sourcing organic products. This makes Carrefour the first retailer to use blockchain technology for their own-brand organic products. The technology will ensure secure data storage and provide transparent access to information about the production and shipping of Carrefour Bio products, from production to store shelves [31]. Finally, in 2023, VeChain, a blockchain supply chain management platform, joined forces with the China Animal Health and Food Safety Alliance (CAFA). This partnership aims to improve food safety and quality control while providing transparent information to consumers by providing a food traceability certification system for CAFA members, enabling them to track the entire life cycle of food products, from production to distribution and sale. The certification will be granted to products that meet CAFA’s quality standards, helping consumers to make informed purchasing decisions. The collaboration is anticipated to boost consumer confidence in Chinese food products and strengthen CAFA’s position in the industry [32].

Overall, the current state of research on the applications of blockchain technology for traceability in the food industry is characterized by a growing interest in the potential of blockchain to improve transparency and trust in the food supply chain. While several challenges and limitations are associated with blockchain adoption, ongoing initiatives and research address these issues and explore blockchain’s full potential in the food industry. Future research could focus on addressing the technical, economic, and regulatory issues associated with implementing blockchain technology in food supply chains and the potential for blockchain to enhance food safety, supply chain efficiency, and consumer trust.

## 3. The Table Olives Supply Chain: Challenges and Opportunities for Implementing Blockchain Traceability Applications

The olive industry is a significant component of the food supply chain, and the traceability of olives is essential to ensure food safety and quality, as the contamination of olives with pesticides, fungi, and bacteria can pose serious health risks to consumers. According to the International Olive Council, the global production of table olives in 2022 was approximately 2.85 million metric tons. The top producers of table olives in 2022 were Spain, Egypt, Turkey, Algeria, and Greece [33].

The table olives supply chain consists of several stages that involve different actors and processes, ranging from the cultivation and harvesting of olives to the final packaging and distribution of the products to consumers. Each stage involves multiple actors, such as farmers, traders, processors, distributors, and retailers, making the traceability of olives challenging. The current system of tracing olives typically relies on paper-based records, which can be lost, damaged, or falsified, leading to inaccuracies and delays in tracing products. Each stage of the supply chain presents specific challenges and opportunities for traceability and quality control, which can be addressed through the use of blockchain technology [34]. However, several challenges are associated with implementing blockchain traceability systems in the table olives supply chains. These challenges can include technical, economic, and social factors that can affect the adoption and effectiveness of the technology. One of the main technical challenges is the interoperability of blockchain platforms and data standards. Several blockchain platforms are available, each with its own technical architecture and features. In order to enable seamless data exchange and integration across different platforms, a common set of data standards and protocols is required. This can be challenging to achieve in the table olives supply chain, where different actors and organizations may use different software systems and data formats [35].

Another technical challenge is the scalability and performance of blockchain systems. Blockchain technology relies on a distributed network of nodes to validate transactions and maintain the ledger. As the number of transactions and users increases, the system may become slower and less efficient, leading to delays and higher transaction costs. This can be a concern in the table olives supply chain, where large volumes of data and complex supply chain interactions may require high-speed and low-cost processing. An economic challenge is the cost of implementing and maintaining blockchain systems. While blockchain technology has the potential to reduce costs and improve efficiency in the supply chain, it also requires significant investment in infrastructure, software development, and training. This can be a barrier to adoption for small and medium-sized enterprises in the table olives supply chain, who may lack the resources and expertise to implement and operate blockchain systems [8].

A social challenge is the need for trust and collaboration among supply chain actors. Blockchain technology can enable greater transparency and accountability in the supply chain, but it also requires a high level of trust and cooperation among the actors involved. This can be a challenge in the table olives supply chain, where there may be issues of power imbalance, information asymmetry, and lack of communication among producers, processors, distributors, and retailers. Building trust and promoting collaboration among these actors is essential for successfully implementing blockchain traceability systems in the table olives supply chain [36].

Despite these challenges, implementing blockchain technology in table olives supply chains can offer several benefits. By providing a secure and transparent record of the product journey, blockchain technology can improve the efficiency and sustainability of the table olives supply chain while enhancing consumer trust and confidence in the products. Moreover, blockchain provides a transparent and immutable record of all transactions, enabling stakeholders to trace the movement of table olives from farm to fork. Additionally, blockchain enhances the trust and transparency of the food supply chain by creating a decentralized network of actors who can verify the authenticity and quality of table olives. Finally, blockchain can facilitate information sharing among stakeholders, enabling real-time monitoring and control of the supply chain [37].

**Figure 1:**
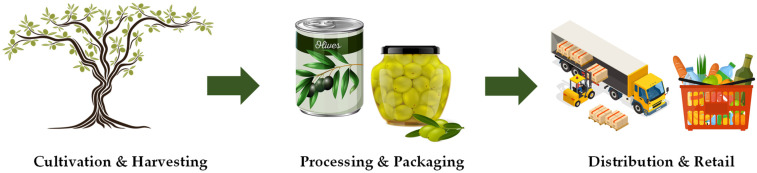
Major Table Olives Supply Chain Stages.

More specifically, the benefits can be identified by analyzing the major table olives supply chain stages, as shown in Figure 1. Each stage presents specific challenges and opportunities for traceability and quality control, which can be addressed through the use of blockchain technology. The first stage of the table olives supply chain is the cultivation and harvesting of olives. This stage involves a range of activities, such as selecting the appropriate olive cultivar, planting and maintaining the trees, and harvesting the olives when they reach maturity. These activities are typically carried out by smallholder farmers or large agricultural enterprises, depending on the production scale. Traceability in this stage can be challenging, as the origin and quality of the olives may be difficult to verify, especially in cases where olives are sourced from multiple suppliers [38].

The second stage of the table olives supply chain is the processing and packaging of olives. Once the olives are harvested, they are typically processed to remove the bitter taste and improve their flavor. This may involve various techniques, such as soaking the olives in water or brine, fermenting them, or flavoring them with herbs and spices. After processing, the olives are packed in containers such as jars or cans, which are labeled with product information such as the origin, date of production, and ingredients. Traceability at this stage can be improved through the use of blockchain technology, which can provide an immutable record of the origin and processing of the olives.

The third stage of the table olives supply chain is the distribution and sales of the table olives. This stage involves transporting the products from the packaging facilities to the retail stores, where they are sold to consumers. The distribution stage can be challenging for traceability, as the products may pass through multiple intermediaries, such as wholesalers and distributors, before reaching the final consumer. However, blockchain technology can enable real-time tracking of the products throughout the distribution chain, providing greater transparency and reducing the risk of fraud or counterfeiting [39].

In summary, implementing blockchain traceability systems in the table olives supply chain involves several technical, economic, and social challenges. Overcoming these challenges requires a comprehensive and collaborative approach that addresses the specific needs and priorities of the different actors involved. By addressing these challenges, blockchain technology can enable greater transparency, efficiency, and sustainability in the table olives supply chain while enhancing consumer trust and confidence in the products. The present research aims to overcome the aforementioned challenges and make use of the opportunities. The following sections present the materials and methods used to develop a simple yet very effective and cost-efficient Ethereum-based distributed application that can enhance and secure the traceability of the table olives supply chain. Afterward, the practical use of the developed application at a Greek table olives producer is demonstrated, and finally, the case study results are analyzed.

## 4. Materials and Methods

As with any technological development solution, it is important to create a business model that extensively describes the solution’s core activities, resources, value proposition, cost structure, revenue streams, and all other elements with which the implementation becomes functional and financially viable. It is also particularly important to take advantage of the opportunities that blockchain presents so that they are contrasted with the problems that this particular application intends to cover. The Ethereum means of implementing the concept of smart contracts that it supports enables its users to create decentralized applications. This is why Ethereum-based distributed applications have been widely used in many industry sectors in recent years. This section presents the methodological framework, which can help anyone aiming to implement a decentralized application on the Ethereum blockchain network. It also describes the functionality and some indicative tools for developing decentralized applications.

In order to develop a decentralized Ethereum application, it is first necessary to create an account or wallet. By creating an account, one can carry out various transactions, either on a testnet, or on the main network of Ethereum. In this way, depending on the used network, coins have to be used for any transaction. During the development and control stage, faucet coins simulate the real Ethereum coins known as “Ethers” without real value. In this way, one can simulate real conditions in order for the programmer to understand the cost of each transaction. However, real Ethers are used for each transaction once deployed on the main network. MetaMask is probably the most popular cryptocurrency wallet that allows one to interact with the Ethereum blockchain. It was created by ConsenSys Software Inc., located in New York, NY, USA, a blockchain software company focused on Ethereum tools. It is an add-on (plugin) available in web browsers as well as in mobile applications. It is essentially a handy and fast means of using decentralized applications since it can sign transactions, send and receive Ethereum coins, and manage account data. The big advantage it offers is the fact that with its use, access to these applications does not require running the full Ethereum node on the user’s machine. In the development part of a smart contract and, by extension, a decentralized application, MetaMask is used so that the developer can perform all needed transactions.

The second step concerns installing a development framework. Truffle is a protocol suite and one of the most renowned development environments used worldwide to develop decentralized applications. It is a testing framework and a means of communication with blockchains since it uses the Ethereum Virtual Machine (EVM), aiming to make a developer’s job easier. With Truffle, the developer gains a toolset that offers the ability to convert the smart contract into machine language (compile), deploy it on any public or private network, connect it to other contracts, and manage its binary code. It provides automatic testing of contracts for very fast development, and it communicates and simulates the Ethereum network locally on the computer, offering the possibility of running transactions at no real cost.

The third step concerns writing the smart contract through available programming languages such as Solidity. Solidity is a high-level, object-oriented programming language, and the Ethereum language source code itself is written in Solidity. This language runs on the EVM and helps scale that machine. It was mainly influenced by C++, Python, and JavaScript. It has a similar syntax to JavaScript, making it easy to understand and approachable for many developers. It is a statically typed language and offers some advantages, such as the inheritance it supports as well as the libraries it has, as it makes it possible to create reusable code, which can be called from different contracts. In order to write a smart contract, numerous tools can be used, even a simple text editor. One of the most frequently used tools is Visual Studio Code (VS Code), with which the developer can give substance to his idea by writing the code of the smart contract he wants to develop in the Solidity programming language. Then, it is required to compile this code into the “EVM bytecode” machine language so that the Ethereum digital engine can understand it. Using the compiler provided by VS Code, the smart contract is converted to machine language. To develop the code, it is also necessary to install and use the web3.js JavaScript library, which is the interface between the web application and the Ethereum blockchain. Another essential element is the code’s Application Binary Interface (ABI), which enables the application to interact with the deployed smart contract. It is a JSON format file that contains all the features and functions of the smart contract.

The fourth step is deploying the smart contract to a test network and testing it. For this research, the Ganache tool is used. It is one of the most accessible tools to use and is recommended by Truffle itself. It is essentially a personal blockchain for development on Ethereum that runs locally on the computer. It is part of the Truffle suite and dramatically simplifies the development of a decentralized application. Ganache automatically makes all the necessary network settings and provides the user with ten accounts holding 100 ETH each and their corresponding mnemonics. It thus manages to simulate the entire vast Ethereum network. Using Ganache, the user can easily check the operation of smart contracts and drill down into details regarding the accounts, balance, and costs required for transactions. Therefore, the developer can experiment with using the balances of the ten accounts and simulate exactly how they want the operation of the smart contract, without spending money and with much faster transaction execution speeds compared to those of the mainnet and public testnets.

The fifth step concerns creating the frontend user interface (UI) of the application. The Truffle suite provides several packages, including functions related to the application’s frontend for the development of the UI. One of these packages is React Box. Using this, the UI of the application is implemented based on the React.js library. This is an open-source library of JavaScript for developing interactive web applications. In the implementation of the frontend part of the decentralized application, the web.js library and the ABI of the contract must be integrated to interact between the smart contract and the web application. In addition, the address of the contract must be embedded in order to invoke its functions, which can be performed automatically through Truffle, without the developer having to do it manually.

The sixth and final step concerns deploying the whole distributed application on the Ethereum mainnet. With this step, the application is now available to the public. However, for it to be available to everyone, the application must be converted so that it can be deployed on a public network such as the mainnet with the help of an Ethereum client. The developer must also have real Ethers in their account. This can happen using a specific infrastructure for deployment, which is essentially a gateway and a web3 provider called Infura. The developer can transfer the local executable they have created locally to the main Ethereum network through the features it provides. After it is deployed, it is sufficient to update the contract account address in the frontend part of the application. Finally, it is required to deploy the frontend part of the application on a server or a Peer to Peer (P2P) network to be fully decentralized. After the deployment, various blockchain explorers can be used for monitoring. Etherscan is the leading explorer for blockchains, an analytical platform for the public data provided on Ethereum. It works in the same way as a search engine through which the user can monitor the Ethereum blockchain in real time, receiving information about transactions, blocks, wallet addresses that are traded, smart contracts, non-fungible tokens (NFT), and many more.

**Figure 2:**
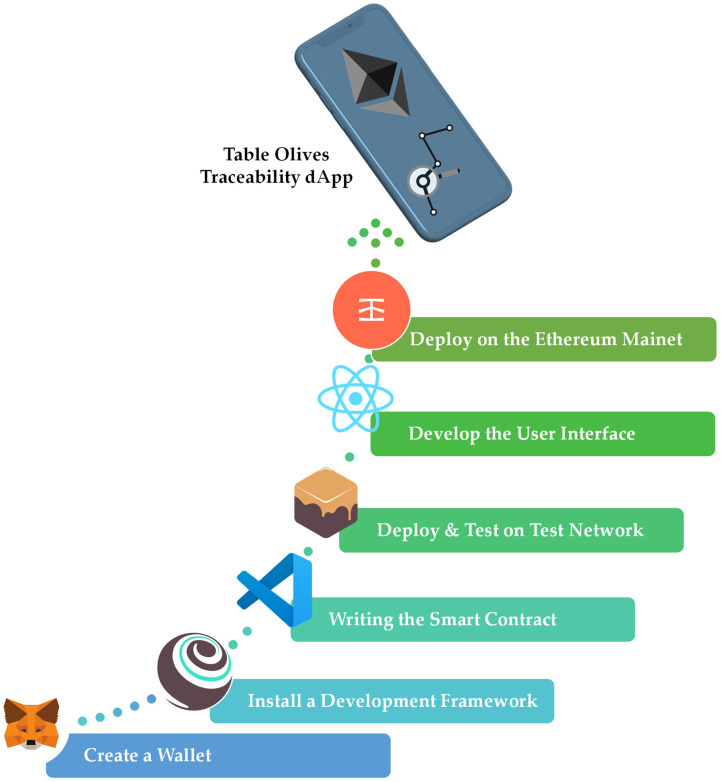
Methodology for Developing an Ethereum-based Distributed Application.

Figure 2, as seen below, summarizes all the steps required to develop an Ethereum distributed application and shows some of the tools used for developing the application of this research’s case study.

The application was developed following the aforementioned methodology. The architecture of the application consists of the three main Ethereum dApp layers being:

1. Application layer: This layer includes the user interface of the dApp and the business logic that interacts with the smart contract. The user interface can be a web application or a mobile application that communicates with the Ethereum network using an API.
2. Smart contract layer: This layer contains the code that defines the rules and logic of the dApp. Smart contracts are written in the Solidity programming language and deployed on the Ethereum network. Once deployed, they are executed by the Ethereum Virtual Machine (EVM).
3. Ethereum network layer: This layer includes the nodes that run the Ethereum software and validate transactions. These nodes form a decentralized network and ensure the integrity of the blockchain.

Additionally, the process of creating and verifying transactions in the Ethereum dApp involves the following five steps based on the Proof-of-Stake consensus mechanism:

1. A user creates a transaction by sending a request to the dApp. The request includes the details of the transaction, such as the amount of Ether to be transferred or the function to be executed in the smart contract.
2. The transaction is created, the user signs it with their private key. This ensures that only the user who owns the private key can authorize the transaction.
3. Participants of the network called “validators” lock up set amounts of ETH in order to obtain a chance to validate the new transaction.
4. A validator is selected to create a block containing the transaction and add it to the network. The likelihood of being selected is proportional to the amount of ETH they have staked and the period for which the ETH have been staked.
5. Other validators in the network validate the block, and if the block is deemed valid, the initial validator earns a reward. However, if the validator fraudulently marks invalid data as valid, they may lose some or all of their stake as a penalty.

## 5. Case Study: Using the Distributed Traceability Application at a Greek Olives Producer

To illustrate the potential benefits of blockchain technology in the table olives supply chain, we present a case study where the distributed application is tested in practice and in real-life conditions on a Greek table olives producer. The olives producer implemented the developed dApp to trace the movement of olives in his entire supply chain, from primary production to consumer sales. The table olives producer currently relies on a manual traceability system facing several challenges that can negatively impact their operations and supply chain. With this system, the table olives producer has to enter data by hand, and in most cases, not in real time, which is time-consuming and prone to errors. Since the system fully relies on human input, there is a great risk of errors and inconsistencies in the data. This leads to delays in tracking and tracing the products, as well as potential mistakes in recording information. Additionally, there is limited data storage capacity, making it difficult to store and access data over time. This can make it challenging to maintain accurate records for the table olive producer’s products and supply chain. Finally, it is impossible to access information about the location and on-time status of table olives. Essentially, traceability is performed by phone calls or emails with other supply chain stakeholders making it challenging to respond quickly to any issues that arise, such as a recall or a quality issue.

**Figure 3:**
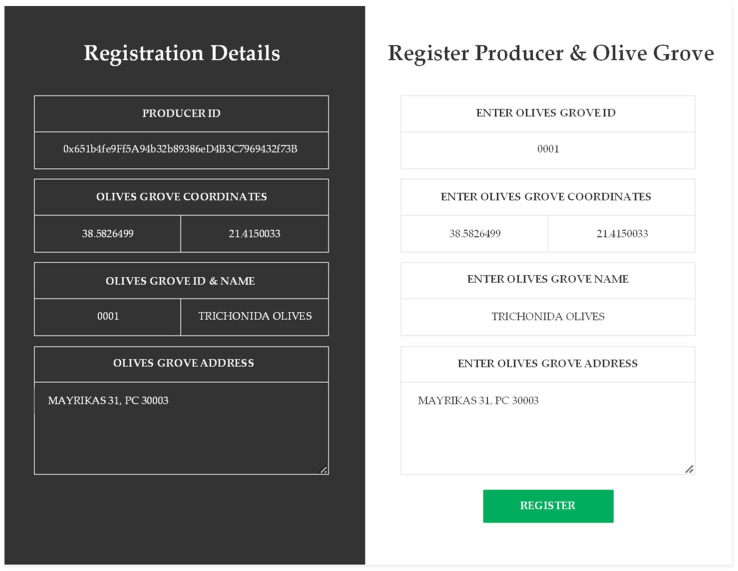
Registration of the Producer and the Olive Grove.

The application involves the use of codes on the packaging of table olives, which consumers can scan to obtain information about the olives’ origin, quality, production, distribution, and sales. For all the involved roles, it was necessary to create MetaMask wallets for making transactions through the dApp. This case study’s first step was registering the table olives producer and the olive grove. All necessary information is entered through the browser UI. More specifically, the entered information concerns the unique number that reflects the olive grove, the location in geolocation format, the name of the olive grove where the harvest took place, and its physical address. When we press the register button, the transaction dialog appears in the producer’s wallet in order to approve it and pay the corresponding price in Ethers. Then the registration must be approved by the administrator of the dApp, and afterward, the user is registered as a producer. After the registration is successful, the contents of Figure 3 are displayed. With the transaction’s approval, and after it has been executed successfully, all the information we entered for the olive grove and the producer’s unique identifier, which is nothing more than the address on the network, appears in the respective fields. In the present case, it is: “0x651b4fe9Ff5A94b32b89386eD4B3C7969432f73B”.

**Figure 4:**
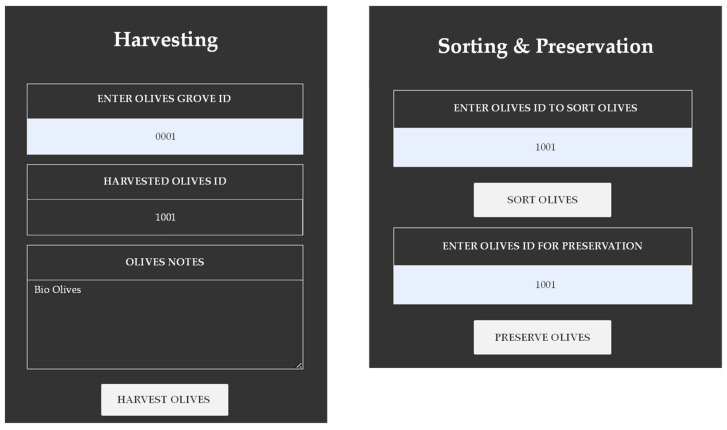
Harvesting, Sorting, and Preservation of Olives.

The next step is assigning all the process fields for the stages followed by the producer. Included are steps such as harvesting the olives from the olive trees, sorting the olives (which ones meet the criteria of the producer and which ones do not), and the maintenance in a container with brine and water for preservation purposes. A unique olives identifier is automatically generated after pressing the “Harvest Olives” button. Therefore, by assigning the unique identifier of the olives and pressing the corresponding button for each stage mentioned, the corresponding transaction is created in the network by removing the corresponding Ethers from the producer’s account. Additionally, for each process, a date and time stamp is recorded automatically by the application. The above screens are seen in Figure 4.

**Figure 5:**
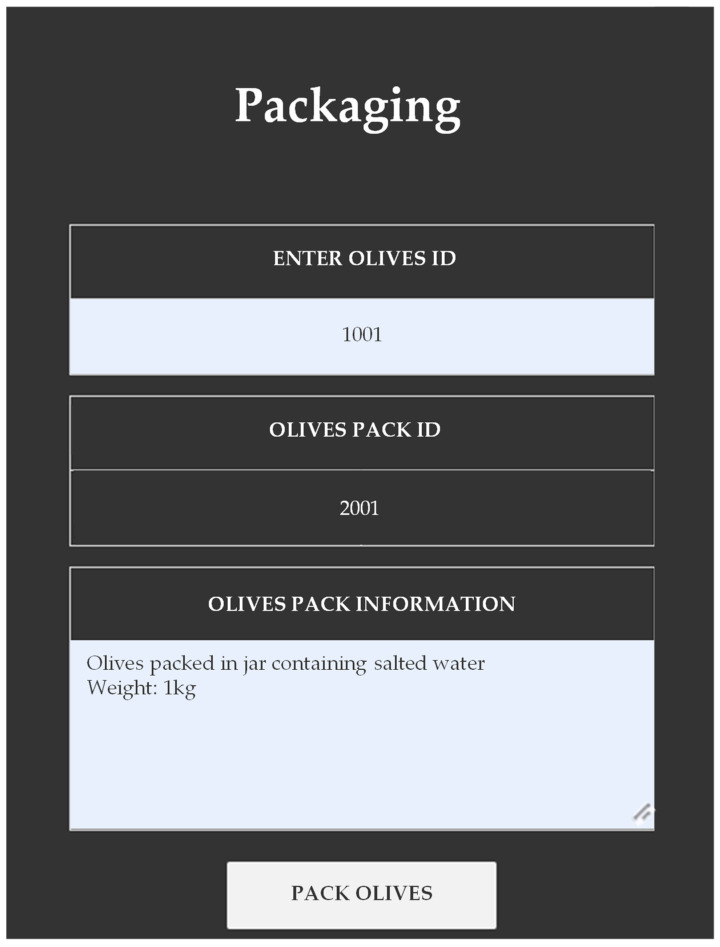
Packaging of Olives.

After assigning all the information about the harvesting, sorting, and preservation stages, the producer enters the unique identification (ID) code of the specific batch of olives given in a previous step (Olives Id) and enters the pack’s information. The app generates a unique code (Olive Pack Id) representing the bottling in a jar, can, or sack, as seen in Figure 5. Once again, the application automatically records a date and time stamp.

**Figure 6:**
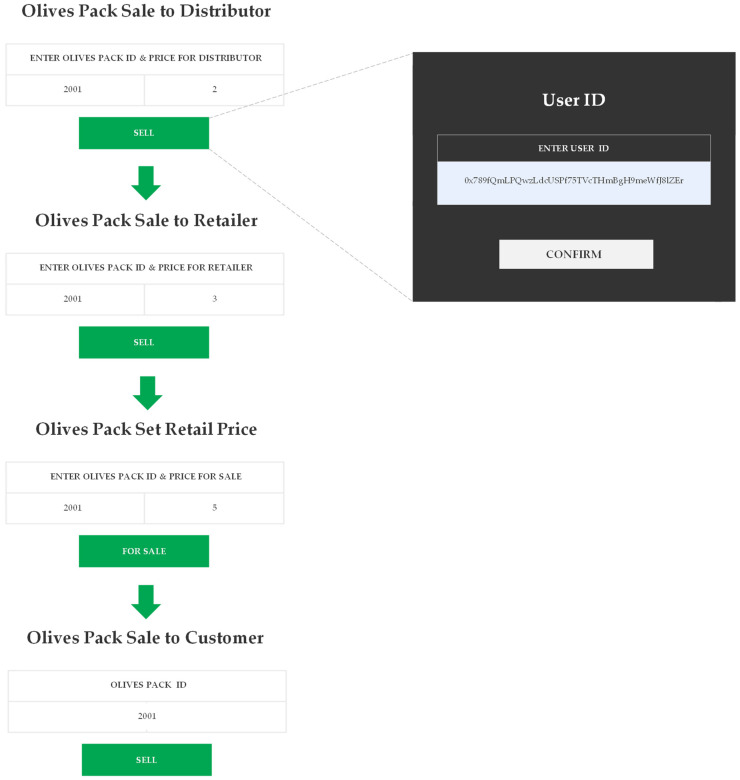
Selling to Distributor, Retailer, and Customer.

In the next step, as seen in Figure 6, the producer assigns the necessary fields in order to sell the packed olives to the distributor based on the unique pack code assigned in the previous step. After purchasing the pack, the distributor becomes the new owner of the product. Then, the distributor can, in turn, sell the corresponding pack of olives to the retailer, and the corresponding transaction can be processed in his wallet. After completing the previous step, the retailer becomes the new owner and can enter the product’s retail price for the consumer. Finally, the retailer can sell the corresponding pack of olives to the consumer, who becomes the final owner of the product. In all previous cases, the application records a date and time stamp when a transaction is performed. Moreover, when the olive packs are sold, a pop-up window opens requiring the seller to enter the buyer’s user ID.

**Figure 7:**
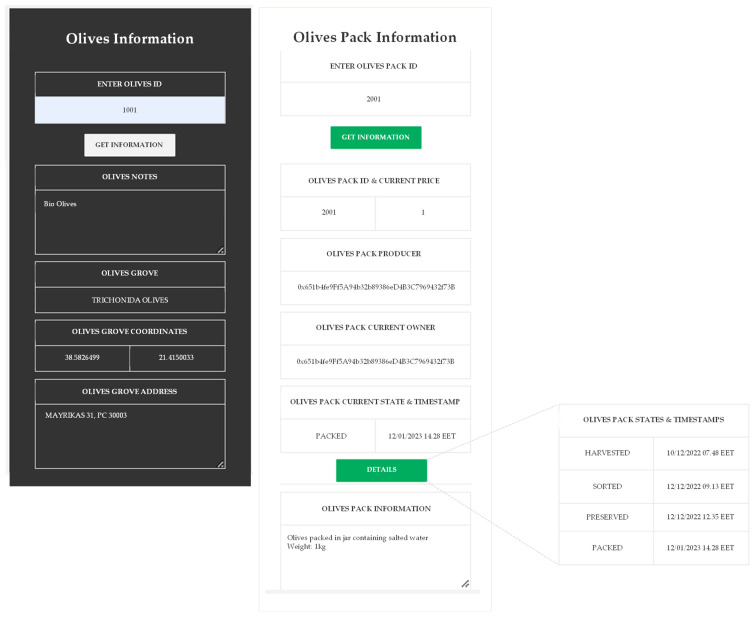
Tracking of Table Olives.

Finally, with the “Olives Information” and “Olives Pack Information” screens, seen in Figure 7, each interested user can gather information about both the olives and the final bottled product regardless of role. The information concerns the state of the product, for example, if it has been harvested, sorted, packed, sold, etc., and the addresses of the producer and current owner. Access to the information is given by entering the pack’s ID. Moreover, by entering the Olives ID, more information can be given about the origin of the olives. For example, in Figure 7, the pack with ID “2001” has been successfully packaged but not yet sold to a distributor. By clicking on the “Details” button, the user can see the complete history of state changes.

## 6. Results

As this paper presents a practical case study, the results section focuses on the practical use of the developed application at the Greek table olives producer. The developed application enabled the producer to achieve full traceability of their products, from when they were harvested to when they were delivered to the end consumer. The application provided a decentralized, secure, and transparent platform where all stakeholders involved in the production and supply of table olives could record and track all the necessary information related to each stage of the supply chain. The application was designed to be user-friendly and accessible to all stakeholders, regardless of their technical skills. The data entered in the application were immutable, meaning that once recorded on the blockchain, they could not be altered or deleted. This ensured the integrity and authenticity of the data, which is critical for ensuring the safety and quality of food products. The application also enabled the producer to quickly identify any issues or irregularities in the supply chain, which could be addressed promptly. This helped to ensure the safety and quality of the products, which is essential for maintaining the producer’s reputation in the market. Overall, the practical use of the developed application demonstrated the potential of blockchain technology for enhancing and securing traceability in the food supply chain. Therefore, by providing a decentralized, secure, and transparent platform, blockchain technology can help improve food products’ safety and quality and strengthen consumer trust in the food industry.

The previous method used by the Greek table olives producer for traceability was a manual system, which was time-consuming, inefficient, and error-prone. The producer had to keep paper records of the entire production process, from cultivation to harvest, sorting, packaging, and distribution. This resulted in a lack of transparency, making it difficult to pinpoint the exact source of a problem in case of a recall. Moreover, the producer had to rely on the trust of intermediaries, which introduced the risk of tampering with the product or its documentation. In contrast, the developed application on the Ethereum network enabled the producer to track and trace the olives’ journey from production to consumption securely and efficiently. Through blockchain technology, the app provided a tamper-proof and decentralized ledger that was accessible to all stakeholders in the supply chain, ensuring transparency and trust. The producer could input and manage all production data, including harvesting, packaging, distribution, and sales information. It should also be noted that the data required for the traceability of the table olives are not confidential so there is no reason for skepticism towards sharing the data.

With the new traceability process, the time required to trace a pack of table olives has been reduced by 98%. Previously, tracking a pack of table olives would take an average of 45 min, as various communications and searches were necessary. With the new application, it takes less than 1 min. Additionally, as the system runs on mobile devices and does not require extensive computing power, data entry can be performed almost in real time and from wherever the user is. Finally, the new traceability application has also resulted in improved customer satisfaction. Customers can now access detailed information about the origin and quality of the table olives they purchase, increasing their trust in the product and the producer.

As a result, the developed application improved the producer’s traceability system in several ways. The application increased the accuracy and reliability of the data, reducing the risk of human error by 67%. Additionally, it provided stakeholders instant access to relevant information, improving the supply chain’s efficiency and responsiveness. Finally, the application helped the producer comply with international regulations and standards, such as the European Union’s General Food Law and Hazard Analysis Critical Control Points (HACCP), which require traceability and transparency in the food supply chain.

## 7. Discussion

Implementing blockchain technology in the food industry is a promising solution to the issues of traceability, transparency, and food safety issues. In this paper, the authors focused on the table olives supply chain, which is one of the most essential products of the Mediterranean diet. The authors analyzed the challenges and opportunities for implementing blockchain traceability systems in this supply chain and developed a methodological framework to help anyone aiming to implement a decentralized application on the Ethereum blockchain network. The research showed that there is a growing interest in blockchain technology by private companies, as well as researchers, to enhance traceability and food safety in the food industry. Furthermore, several pilot programs are in progress, and many research publications aim to test the feasibility of blockchain-based traceability systems in various food products.

In the present study, a distributed application for table olives’ traceability on the Ethereum network was developed, demonstrating blockchain technology’s potential in securing the food supply chain. The application allowed for the traceability of table olives, from the producer to the consumer, through a transparent and immutable ledger. It also provided an easy-to-use interface for all users, from producers to retailers, to interact with the blockchain and record their transactions. The developed application for table olives’ traceability on the Ethereum network significantly improved the Greek table olives producer’s traceability system. The application provided a secure, transparent, and efficient solution for tracking and tracing the products in the supply chain. The app reduced the time, increased the accuracy and reliability of data, improved supply chain efficiency, and helped the producer comply with international regulations and standards.

Despite the potential benefits of blockchain technology in enhancing traceability and transparency in the food industry, some limitations to its implementation are also valid limitations of the present research. One of the main challenges is the cost of implementing and maintaining the blockchain-based application, which can be a significant barrier for small-scale producers or low-income countries. It should be noted that despite the relatively high cost of implementing and maintaining such an application, such implementations can significantly reduce supply chain costs in cases such as the need for intermediaries, and product recalls. In order to effectively cope with this challenge, there is a need for governmental and societal pressure at a global level to accelerate adoption and implementation. Governments can play a crucial role in promoting the adoption of blockchain food traceability systems by implementing policies that encourage the use of this technology. For example, governments can provide financial incentives for companies that implement blockchain food traceability systems, or require companies to implement these systems as part of food safety regulations. By doing so, governments can create a level playing field for companies that implement blockchain technology and promote a culture of transparency and accountability in the food supply chain [40].

Similarly, societal pressure can also play a role in driving the adoption of blockchain food traceability systems. Consumers are increasingly concerned about the safety and quality of their food, and blockchain technology can help to address these concerns by providing transparency and traceability throughout the supply chain. By raising awareness of the benefits of blockchain food traceability systems and advocating for their implementation, consumers can put pressure on companies to adopt this technology. Finally, initiatives such as international collaborations and partnerships can also play a significant role in promoting the adoption of blockchain food traceability systems. These collaborations can bring together stakeholders from across the food supply chain to work together towards implementing blockchain technology and creating a more transparent and efficient food supply chain. These collaborations can also reduce the cost for such implementations by creating economies of scale.

Another limitation is the need for standardization and interoperability between different blockchain platforms to ensure that data can be shared and integrated across different supply chains. Additionally, a significant limitation of blockchain technology is scalability. As more transactions are added to the blockchain, the size of the blockchain grows, and the time it takes to process each transaction increases. This can make it challenging to implement a blockchain-based traceability application at a large scale, where there are many participants and a high volume of transactions. Various scaling solutions have been proposed to address this issue, such as off-chain transactions, sharding, and sidechains. These solutions aim to reduce the load on the blockchain network and increase its capacity to handle a larger number of transactions. However, these solutions are still in development and require further research and testing to ensure their reliability and efficiency.

Another issue related to the blockchain-based traceability application is the accuracy and consistency of user data entry. As the application relies on input from various participants along the supply chain, there is a risk of errors or intentional data falsification. This can undermine the application’s integrity and compromise its effectiveness in enhancing traceability and transparency. To address this issue, it is essential to establish clear guidelines and protocols for data entry, to ensure that all participants understand the importance of accurate and consistent data input. This can be supported by the use of automated data collection and monitoring tools, such as sensors and IoT devices, which can provide real-time data and reduce the reliance on manual data entry. Moreover, using data validation mechanisms, such as cryptographic algorithms and smart contracts, can also help to ensure the data’s integrity and prevent the records’ tampering or manipulation.

There is still much potential for using blockchain technology to enhance traceability and transparency in the food industry. However, in order to manage to implementation of blockchain technology on a wide scale, it is necessary to convince food supply chain actors about its benefits. One reason why food supply chain actors may be hesitant to adopt blockchain technology is a lack of understanding of how the technology works and how it can benefit their operations. Therefore, there is a need to educate stakeholders on the potential benefits of blockchain technology, including increased transparency, traceability, and efficiency. This education can take the form of case studies, demonstrations, and workshops that highlight the practical applications of blockchain technology in the food industry. Another reason why food supply chain actors may be hesitant to adopt blockchain technology is concerns about the security of the technology. Given the sensitive nature of food supply chain data, there is a need to reassure stakeholders about the security of blockchain technology and the measures in place to protect their data. Finally, there may be concerns about the cost of implementing blockchain technology in the food supply chain. However, it is important to highlight the potential long-term cost savings associated with the increased efficiency and traceability provided by blockchain technology.

As technology evolves, there is a need for further research to optimize the design and implementation of blockchain-based applications to ensure that they are accessible, cost-effective, and efficient. One potential direction for future research is integrating blockchain technology with other emerging technologies, such as the Internet of Things (IoT), to provide real-time monitoring and tracking of food products along the supply chain. This could also enable the collection of more detailed and accurate data, which can be used for analytics and optimization of the supply chain. Another potential direction is the use of blockchain technology in enabling more sustainable and ethical food production practices by providing a platform for certification and verification of environmental and social standards. This could help to address the growing demand for sustainable and ethically produced food products and provide greater transparency and accountability in the food supply chain.

Moreover, future research on blockchain traceability applications should focus on supporting circular food supply chains and related processes. Recycling of food waste is a critical aspect of sustainable food production and consumption, and traceability applications can play a crucial role in supporting this process and the circular economy in general. In fact, an emerging trend in food research is the recycling of food byproducts into new food products [41]. Blockchain traceability applications can enhance this process by offering a record of the handling and storage of food byproducts throughout the supply chain, including transportation, processing, and storage. In this way, they can help identify the sources of food byproducts, including the type of food, the location of the production facility, and the processing methods used. This information can help determine the quality and safety of the byproducts, as well as their potential uses in new food products. Most importantly, blockchain traceability applications can verify claims about the environmental benefits of using recycled food byproducts and support trust in sustainable food products as the origin and processing of the byproducts can be fully traced and proven.

In conclusion, as is clear from the research, blockchain technology has significant potential to enhance traceability and transparency in the food industry. Further research and development are needed to address the limitations and optimize the design and implementation of blockchain-based systems.

## Data Availability

Data is contained within the article.
